# Mania induced after corticosteroid treatment: a case report

**DOI:** 10.1192/j.eurpsy.2023.1458

**Published:** 2023-07-19

**Authors:** E. Arroyo Sánchez, A. Sanz- Giancola, P. Setién Preciados, I. Romero Gerechter, M. Martín Velasco, C. Díaz Mayoral

**Affiliations:** Psiquiatría, Hospital Universitario Príncipe de Asturias, Madrid, Spain

## Abstract

**Introduction:**

Corticosteroids are drugs widely used in clinical practice for their anti-inflammatory and immunosuppressive properties. Despite their beneficial effects, a high association of these drugs with neuropsychiatric adverse effects such as psychosis, mania, depression, delirium or increased risk of suicide, among others, has been observed. We present the case of 54-year-old man who started treatment with hydroaltesone 20 mg/8h after undergoing surgery for a pituitary macroadenoma who began with maniform clinic.

**Objectives:**

To know the prevalence, risk factors and treatment of mania as a side effect of corticosteroid drugs.

**Methods:**

Presentation of the case and review of the available literature on the risk of developing mania after corticosteroid treatment.

**Results:**

Several studies confirm that the incidence of psychiatric adverse effects after the use of corticosteroids is around 6% if we refer to severe reactions; 28% moderate reactions; and 72% if we consider milder reactions. The direct relationship between these drugs and affective symptoms ranges in rates between 1-50% of cases, the most frequent being depression and mania. The risk of mania after treatment with corticosteroids is 4-5 times higher than if we compare it with a group of population not exposed to these drugs. There is a dose-response relationship, increasing the risk from a daily dose of 40 mg/day, with an average duration of symptoms of around 21 days. Female sex seems to be a risk factor in relation to the fact that diseases requiring this type of treatment are more common in this gender. As first-line treatment for mania secondary to corticosteroids, a decrease in treatment dose or its interruption, whenever possible, is proposed. Adjuvant treatment may be required, with atypical antipsychotics being the first choice.

**Conclusions:**

Corticosteroid therapy has a direct dose-response relationship with the presence of psychiatric adverse effects such as mania. Dose and sex have been studied as possible adverse effects. Therefore, the pharmacological treatment of choice consists of a reduction in the dose of corticosteroids administered or withdrawal, if possible, and may be combined with an atypical antipsychotic such as olanzapine, quetiapine or risperidone. Re-evaluation is recommended until complete resolution of the clinical picture and then antipsychotic treatment can be progressively withdrawn.

**Disclosure of Interest:**

None Declared

